# Fast filtered backprojection algorithm for low-dose computed tomography

**DOI:** 10.14312/2399-8172.2020-7

**Published:** 2020-11-30

**Authors:** Gengsheng L. Zeng

**Affiliations:** 1 Department of Computer Science, Utah Valley University, 800 West University Parkway, Orem, UT 84058, United States of America; 2 Department of Radiology and Imaging Sciences, University of Utah, 729 Arapeen Drive, Salt Lake City, Utah, 84108, United States of America

**Keywords:** low-dose CT, denoising, linear filter, nonstationary filter

## Abstract

Low-dose computed tomography (CT) can produce noisy images that may contain streaking artifacts. Removal of streaking artifacts normally requires iterative algorithms that model the transmission noise physics. A fast filtered backprojection (FBP) algorithm is introduced in this short paper. This algorithm is very simple and effective in removing the streaking artifacts in low-dose CT.

## Introduction

X-ray computed tomography (CT) has been around for more than 45 years [1]. Mainly due to the popularity of the CT scans, Americans’ radiation exposure has risen six-fold in the past 35 years, according to a National Council on Radiation Protection and Measurement report [2]. The increased use of CT scans stems largely from screening CT of the lung in smokers, virtual colonoscopy, CT cardiac screening, CT-based attenuation correction for positron emission tomography (PET)/CT imaging, whole-body CT in asymptomatic patients [3, 4], and CT imaging of children [5]. Shortening of the scanning time to around one second, which eliminates the strict need for the subject to remain still or be sedated, is one of the main reasons for the large increase in the pediatric population. CT scans of children have been estimated to produce non-negligible increases in the probability of lifetime cancer mortality, leading to calls for the use of reduced current settings for CT scans of children. For these reasons, the CT industry has put in a lot of effort to develop low-dose CT. One active area of research is into methods to reduce the radiation counts by applying adaptive collimation to block unnecessary x-ray photons. Another active area of research is into development of more robust image reconstruction algorithms that are less sensitive to noise in low-count data. This paper is focused on the second approach — developing a fast, robust reconstruction algorithm.

Reducing dose causes more noise in the CT image. Sometimes, streaking artifacts appear in the low-dose CT images as well. Many research groups have shown that iterative image reconstruction algorithms can be used to generate less-noisy images than the analytical filtered backprojection (FBP) algorithm with the same data set [6–9]. The FBP algorithms are still useful if the noisy projections are pre-filtered in low-dose CT applications [10–12].

In addition to analytic algorithms and iterative algorithms, most recently the deep learning-based CT image reconstruction methods has been dominating the journals and conferences [13–18]. The deep learning-based methods are able to reduce the noise for low-dose CT images. Duke research group did some comparison studies between the FBP, the iterative algorithms, and GE’s TrueFidelity™ deep learning-based image reconstruction algorithm [13–15] and found the following: The deep learning-based method has similar performance to iterative reconstruction methods in the sense that they demonstrate a locally heterogeneous spatial distribution of noise and reduced low-contrast spatial resolution.

We recently derived some effective FBP algorithms for low-dose CT [19–21]. Our main idea was to use spatially variant linear filter to suppress noise in the projections (i.e., sinogram). In other words, different linear filterers were used for different projections. In reference [19], the filters were in the frequency-domain. Ten filters were designed and applied to the sinogram, resulting in ten versions of the filtered sinograms. Then a combined version was formed according to the noise model. This combined version was used in an FBP algorithm for image reconstruction. In reference [20], attempts were made to convert the frequency-domain filter’s transfer function into the spatial-domain convolution kernel. Approximations were used in kernel calculation. As a result, the spatially variant filter could be implemented in the spatial-domain, without using the Fourier transform. In reference [21], the filters were spatial-domain Gaussian two-dimensional (2D) filters, which were spatially variant.

The current paper suggests a simple one-dimensional (1D) filter for noise control. This filter will be described in the methods section. Clinical image results are presented, and the effectiveness of the proposed filter is demonstrated in the results section.

## Methods

The theoretical background was previously established in [19] based on minimization of a weighted least-squared objective function, and the weighting function was determined by the noise variance. Minimization of this weighted least-squared objective function led to a spatially variant low-pass filter. The frequency-domain transfer function of this low-pass filter was

$$\text{H}(\omega )=1-{\left(1-\frac{\alpha w}{\left|\omega \right|}\right)}^{k}\;and\;H(0)=1,$$

where ω was the frequency in radians, α was a parameter emulating the step-size of an iterative algorithm, k was a parameter emulating the iteration number of an iterative algorithm, and w is the noise-variance dependent weighting factor. This low-pass filter in [19] continuously varied with the projection noise variance.

Here is how the filter in [19] can be quantized and simplified in this paper. When noise is small, αw/|ω| is large and H(ω) is almost an all-pass filter. In other words, no filter is required. When noise is large, αw/|ω| is small and H(ω) is a narrow-band low-pass filter. The low-pass filter is not unique. In this paper, we simplify this narrow-band low-pass filter by eliminating the parameters α and *k*. The simple boxcar filter is chosen in this paper for this narrow-band low-pass filter. The boxcar filter is essentially the *n*-point average filter. The frequency-domain transfer function of this boxcar filter is a sinc function and the bandwidth of the filter is determined by the parameter *n*. A larger *n* corresponds to a narrower bandwidth.

The novelty and significance of the proposed method can be recognized by the fact that the projection ray dependent denoising is normally achieved by using an iterative algorithm, which properly weights each projection ray with a weighting function. An iterative algorithm is much less computationally efficient than an analytic algorithm. There are many analytic algorithms, some are more efficient than others. To the author’s knowledge, the proposed algorithm is the most efficient image reconstruction algorithm with nonstationary denoising.

**Figure 1:**
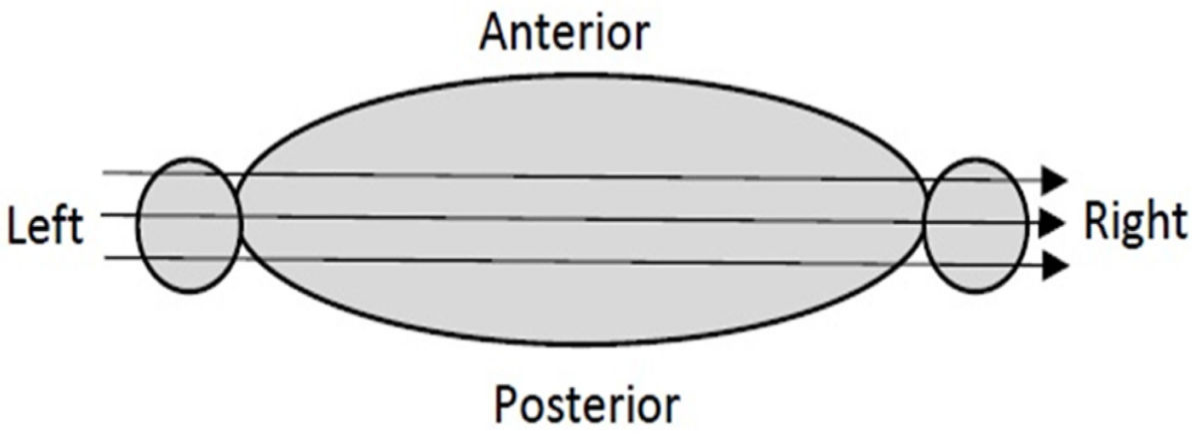
The x-rays passing through both shoulders or both arms have the largest attenuation and thus largest noise.

In x-ray CT imaging, a transmission noise model is assumed, and the noise variance is an exponential function of the ray sum, which is also referred to as line integral, Radon transform, or projection [22]. A larger ray sum is corrupted with larger noise. For human torso low-dose x-ray CT imaging, the ray sums from shoulder to shoulder have the largest values, compared with ray sums in other directions (Figure 1). The x-rays passing through both shoulders or both arms have the largest attenuation. Thus, the projections measured at the views seeing both shoulders or both arms are the noisiest. The ray sum values get even larger if they pass through the bones.

**Figure 2:**
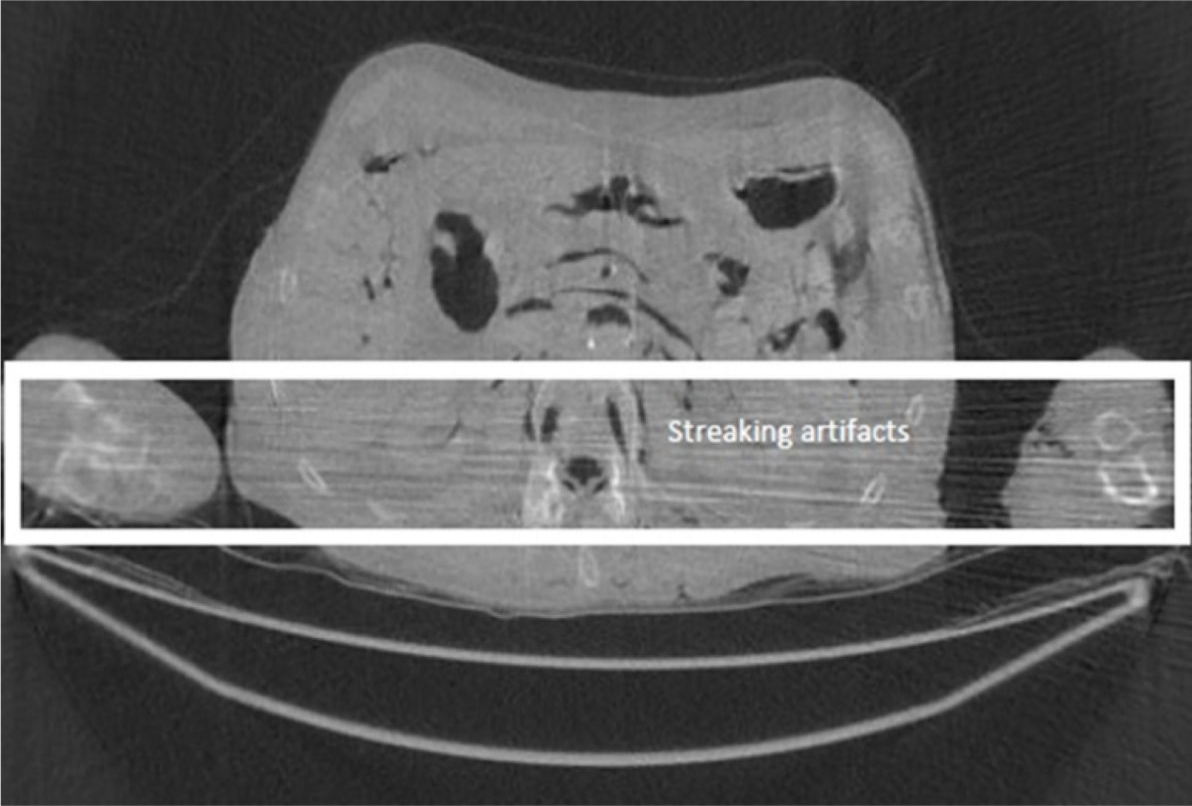
A CT image that suffers streaking line artifacts from arm to arm.

In the FBP algorithm, a ramp filter is applied to the projections before backprojection. The ramp filter is a high-pass filter, and it amplifies the noise in the projections. The backprojection procedure then propagates the data and noise into the image domain. The significant noise from the worst views is amplified by the ramp filter and propagates into the image as streaking lines (Figure 2).

This paper proposes a simple fast filtering scheme to remove the streaking artifacts. The philosophy behind the proposed filtering scheme is as follows. For those projections that do not introduce streaking artifacts, no filtering is required. For those projections that introduce streaking artifacts, we use a fastest and simplest way to denoise. A simple way to separate these two groups of data is to use a threshold value, *T*, which is determined by experience. It is convenient to denote *T* as the percentage of the maximum projection value. The simplest lowpass filter is an *n*-point average, which is defined as the sum of *n* points in the neighborhood (including itself) divided by *n*. The neighborhood size *n* is another parameter determined by experience.

The proposed filtering scheme consists of the following steps:

*Step 1:* Read in projections and find their maximum value. Select a threshold value, *T*, based on the maximum value. Select an odd integer *n*.

*Step 2:* Loop through all projections If projection

If projection< T

new_projection = projection

else

new_projection = 1D *n*-point average of projections

end if

*Step 3*: FBP reconstruction using new_projections.

In the results section of this paper, the effectiveness of the proposed algorithm is evaluated using the Sum Square Difference (*SSD*), which is defined as

$$SSD=\frac{1}{\sqrt{\Sigma_{i,j}{\left[I_{H}(i,j)\right]}^{2}\Sigma_{i,j}{\left[I_{L}(i,j)\right]}^{2}}}\sum_{i,j}{\left[I_{H}(i,j)-I_{L}(i,j)\right]}^{2}$$

where *I*_*L*_ is the image reconstructed using the low-dose data and *I*_*H*_ is the image reconstructed using the regular-dose data. The regular-dose image is treated as the gold standard.

The reconstructed images are also compared using the noise power spectrum image, which is the magnitude image of the 2D Fourier transform of the difference image of *I*_*L*_ - *I*_*H*_. If everything is perfect, this Fourier-domain image is a constant zero. This Fourier-domain image is able to catch the artifacts in the associated spatial-domain image. For example, if the spatial-domain artifacts are the horizontal streaks, they are indicated by bright values long the vertical direction in the Fourier spectrum image.

In this paper, the proposed algorithm is compared with two algorithms: the conventional FBP algorithm and an iterative algorithm that models the CT noise and enforces the image non-negativity [23]. The conventional FBP algorithm is unable to remove the streaking artifacts. The iterative algorithm can significantly reduce the artifacts, while the spatial resolution is degraded slightly.

## Results

In this section, a clinical cadaver torso is used to test our proposed algorithm. A diagnostic scanner (Aquilion ONE™, Toshiba America Medical Systems, Tustin, CA, USA) was used to scan the object (raw data courtesy of Leiden University Medical Center). The scanner used a cone-beam imaging geometry with a fan angle of was 49.2°. The X-ray tube rotated around the object in a circle of a radius of 600 mm, 1200 views uniformly sampled over 360°. The detector of the machine had 320 rows. Each detection row consisted of 896 channels. The row-height was 0.5 mm. The tube voltage was set at 120 kV. The current was set at 500 mAs for the full-dose mode and the current was set at 60 mAs for the low-dose mode. Each cone-beam image volume contained 320 slices. We present results of 3 slices in this section.

**Figure 3:**
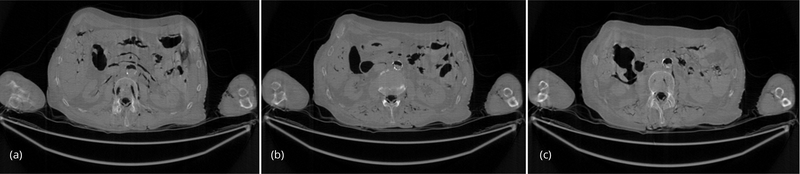
(a) Slice #1 of the regular-dose FBP image. This is the gold standard. (b) Slice #32 of the regular-dose FBP image. This is the gold standard. (c) Slice #64 of the regular-dose FBP image. This is the gold standard.

For the comparison purposes, the regular-dose (instead of low-dose) projections were used to generate 3 artifact-free images, and the 3 artifact-free reconstructed images are shown in Figures 3 as gold standards. These 3 images were reconstructed by the conventional FBP algorithm.

**Figure 4:**
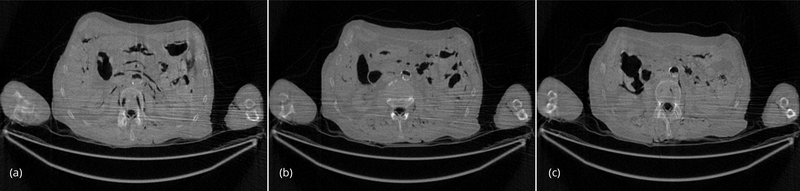
(a) Slice #1 of the low-dose FBP image. This image contains severe streaking artifacts. (b) Slice #32 of the low-dose FBP image. This image contains severe streaking artifacts. (c) Slice #64 of the low-dose FBP image. This image contains severe streaking artifacts.

**Figure 5:**
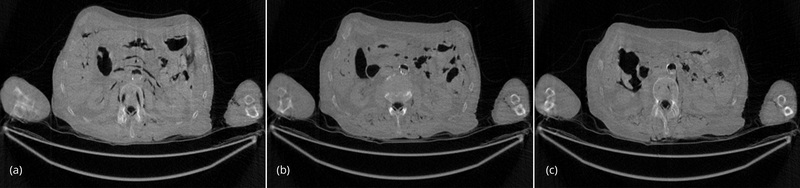
(a) Slice #1 of the low-dose FBP image, using proposed fast denoising filter. The streaking artifacts are removed. (b) Slice #32 of the low-dose FBP image, using proposed fast denoising filter. The streaking artifacts are removed. (c) Slice #64 of the low-dose FBP image, using proposed fast denoising filter. The streaking artifacts are removed.

The conventional FBP reconstructions of the same 3 slices, using the low-dose data, are shown in Figures 4. The horizontal streaking artifacts are clearly seen in these images between the two arms. Figure 5 show the FBP reconstructions of the same 3 slices, using the low-dose data and the proposed denoising algorithm. By comparing Figures 5 with Figures 4, it is clear that the streaking artifacts are successfully removed by the proposed algorithm. The numerical results using the *SSD* distance are shown in Table 1.

**Figure 6:**
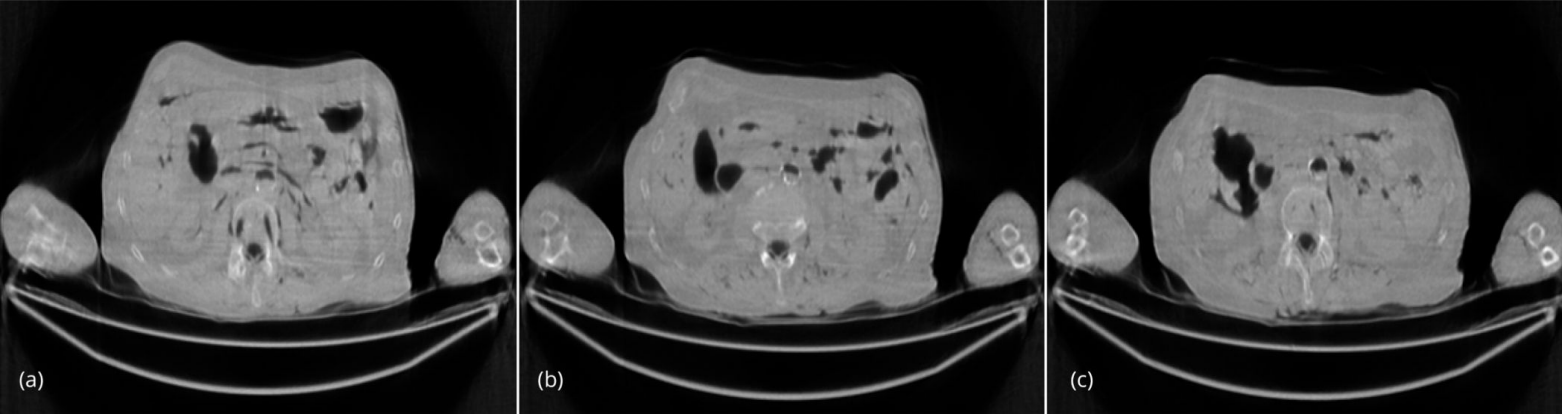
(a) Slice #1 of the low-dose iterative reconstruction, using 200 iterations of the iterative transmission algorithm with noise weighting. The streaking artifacts are significantly reduced. (b) Slice #32 of the low-dose iterative reconstruction, using 200 iterations of the iterative transmission algorithm with noise weighting. The streaking artifacts are significantly reduced. (c) Slice #64 of the low-dose iterative reconstruction, using 200 iterations of the iterative transmission algorithm with noise weighting. The streaking artifacts are significantly reduced.

The reconstructed images with 200 iterations of the iterative algorithm using the low-dose data are shown in Figures 5. The iterative algorithm models the x-ray CT transmission noise and enforces the image non-negativity. Even though the streaking artifacts are significantly suppressed, the image spatial resolution is somewhat compromised. The numerical results using the SSD distance are also shown in Table 1. The reconstructed images with 200 iterations of the iterative algorithm using the low-dose data are shown in Figure 6. The iterative algorithm models the x-ray CT transmission noise and enforces the image non-negativity. Even though the streaking artifacts are significantly suppressed, the image spatial resolution is somewhat compromised. The numerical results using the SSD distance are also shown in Table 1.

**Figure 7:**
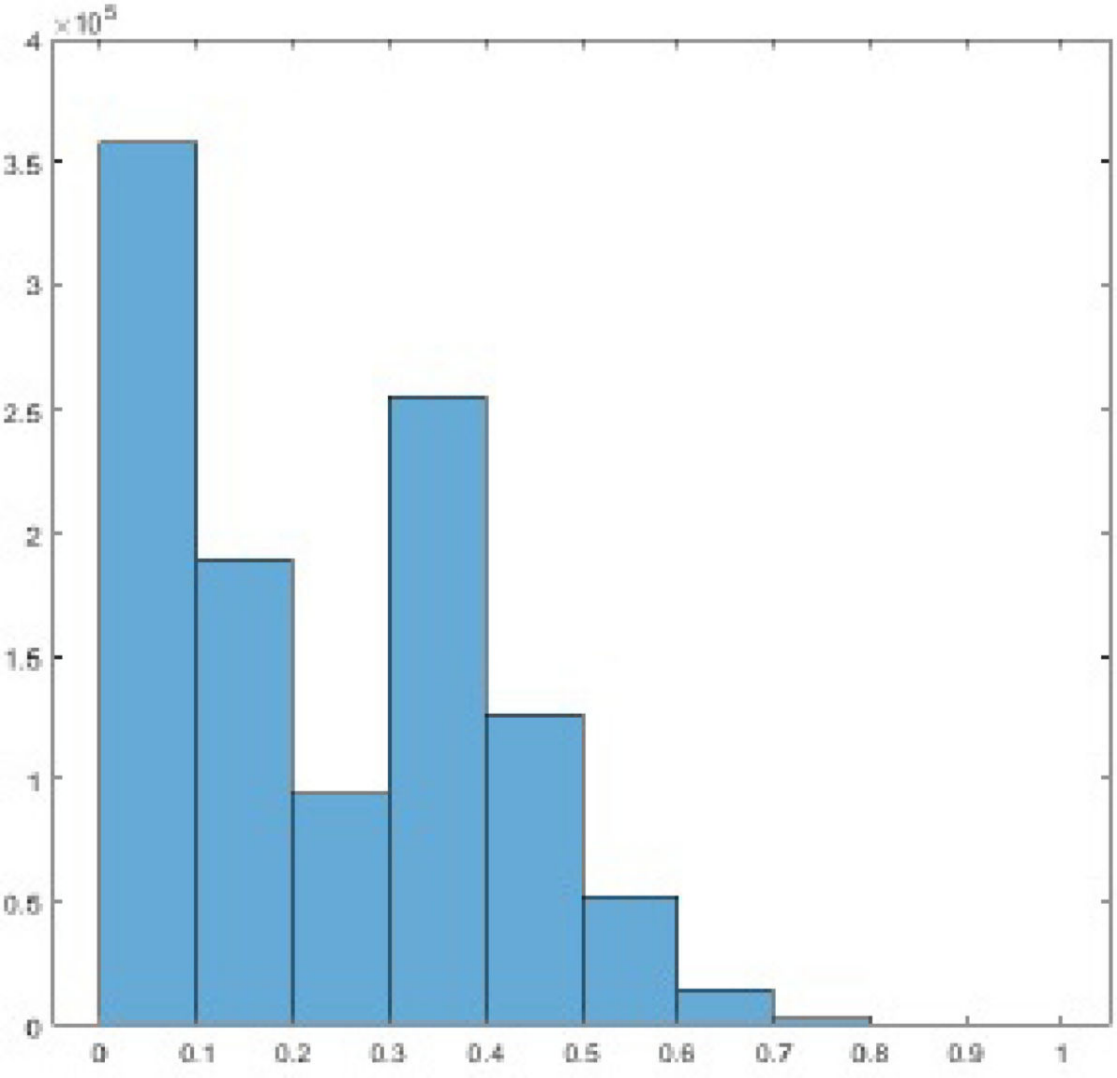
A histogram of the projection sinogram. The value ‘1’ in the horizontal axis indicates the maximum value of the projections in the sinogram.

**Figure 8:**
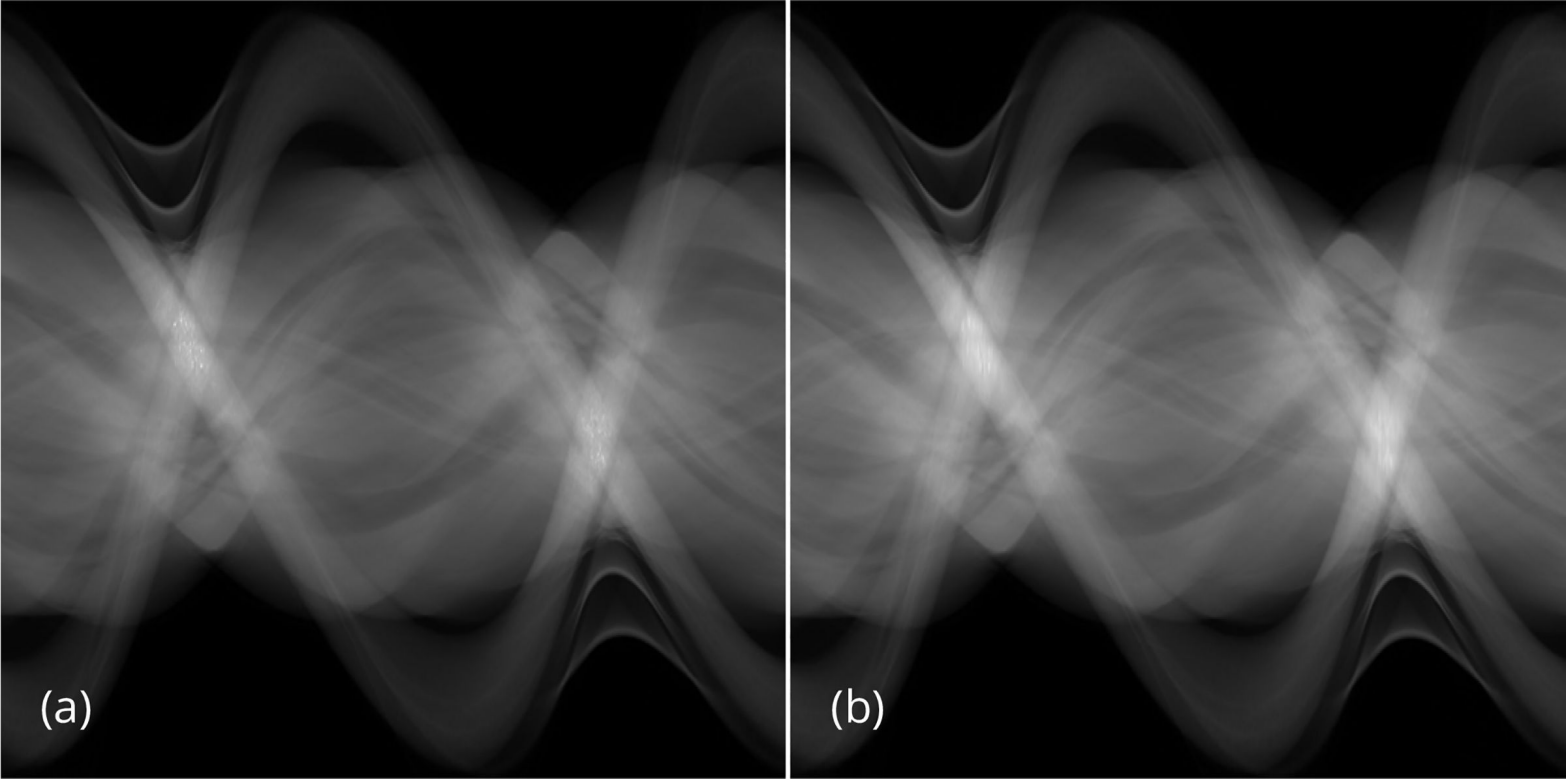
(a) Raw projections for slice #32 before processing. (b) Processed projections for slice #32 using proposed algorithm.

In the implementation of the proposed algorithm, the threshold *T* was set at the 60% of the maximum projection value. The parameter *n* was set as 13. A representative histogram of the projection sinogram is shown in Figure 7. In this histogram, 98.44% of the total projections are below the threshold of 60%; only 1.56% of the total projections are above the threshold of 60%. This fact can be visualized by the projection images (also known as the sinograms) shown in Figure 8. The sinogram difference between before and after pre-filtering only happens to the 1.56% brightest sinogram values. The 98.44% of the sinograms are not affected. The computation overhead beyond the conventional BFP algorithm is negligible. Also, much of the computation time in the FBP algorithm is spent on the backprojection procedure. The total computation time of the proposed algorithm is almost the same as the computation time of the conventional FBP algorithm.

**Figure 9:**
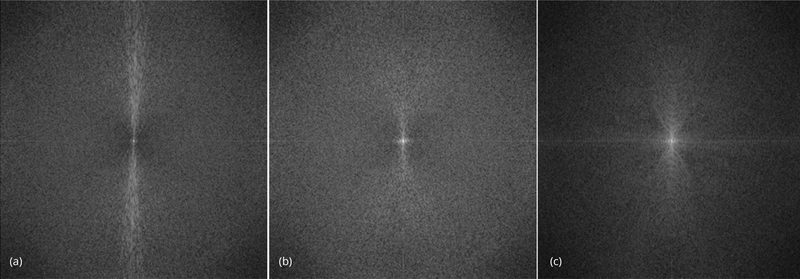
Noise power spectra for slice #32. (a) Conventional FBP reconstruction. (b) Proposed pre-filter method. (c) Iterative reconstruction.

The effectiveness of the proposed algorithm can also be indicated by the noise power spectrum images shown in Figure 9. The horizontal streaking artifacts in the spatial images are represented by the central verticl bright regions, which can be visualized in the noise power spectrum image associated with the conventional FBP algorithm, but not in the other two spectrum images.

## Discussion

One may have a concern that noise suppression by the application of a low-pass filter may blur or remove the lesions, which are important in clinical diagnosis. This concern is valid for the common spatially invariant low-pass filter. If a spatially invariant low-pass filter is applied to a sinogram, all projections will be blurred. As a result, the lesions will be blurred.

On the other hand, the proposed denoising method is spatially variant. A lesion is measured in all views. In the proposed algorithm, the lesion projections are not filtered in most projections. The low-pass filter is used only at a small number of projections, where the noise is the largest. The proposed method reduces the impact of the low-pass filter to its minimum. The performance of the proposed algorithm is demonstrated by the 3 reconstructed slices of real CT scans. Using a cadaver makes it possible to have a gold standard (with a regular x-ray dose) to compare the low-dose images. It is clearly observed that the small objects or shapes are well kept and the sharpness of the edges are unaffected with the proposed spatially variant technique. A spatially invariant denoising filter would blur the entire image (not shown).

## Conclusions

The main motivation of developing the proposed filter is the computational efficiency. The total computation time of the proposed FBP algorithm is almost the same as the conventional FBP algorithm. In the proposed algorithm, no action is required if the ray sum value is smaller than a threshold, *T*, which is selected by trial-and-error. These data are approximately 98% of the total projections. The purpose of this threshold value *T* is to identify the ray sums that may cause the streaking artifacts in the image. Only the relatively large ray sums can contribute to the streaking artifacts.

When the ray sum is greater than the threshold value *T*, a simplest 1D lowpass filter is used to smooth the sinogram. The simplest lowpass filter is the *n*-point average filter, which sums *n* samples and divides the sum by *n*. This value *n* is an odd integer, determined by trail-and-error as well. Fortunately, the filter performance is not sensitive to parameters *T* and *n*. For example, after changing *n* = 13 to *n* =15, the *SSD* for slice #1 using the proposed algorithm changes from *SSD* = 0.0146 to *SSD* = 0.0145. One cannot visually tell the differences between the image using *n* = 13 and the image using *n* =15. If we change the threshold from 60% of the maximum projection value to 65%, the *SSD* improves to *SSD* = 0.0130 for slice #1 with *n* = 15. The proposed algorithm is thus found to be fairly robust and not sensitive to the scanning target. The rule of thumb to select the parameter *T* is that only a very small percentage of the sinogram values are selected to be filtered. This task can be achieved by evaluating the histogram of the sinogram. The selection of the parameter *n* is not critical, as along as the parameter *n* is large enough, almost the same resultant is obtained.

Our proposed algorithm does not need a complicated mathematical noise model. No Bayesian constraints are required. The algorithm is non-iterative. We believe that our algorithm is a cost-effective tool to combat the artifacts due to the data starvation nature of low-dose CT.

## Figures and Tables

**Table 1:** Sum square distance (*SSD*) between the low-dose image and the regular-dose image.

Image slice number	SSD value of the conventional FBP reconstruction	SSD value of the proposed FBP reconstruction	SSD value of the iterative reconstruction
#1	0.0189	0.0146	0.1097
#32	0.0065	0.0053	0.1208
#64	0.0094	0.0048	0.1418
